# 

**Published:** 1909-09

**Authors:** 


### BOOKS RECEIVED.

Lectures on Hysteria and Allied Vasomotor Conditions. By Thomas Dixon Saville, M.D., Lond., Physician to the West End Hospital for Diseases of the Nervous System, and to the Saint John's Hospital for Diseases of the Skin, Leicester Square, London. New York: William Wood & Co. London: Henry J. Glaisher. 1909. (Price, $2.50.)

Manual of the Diseases of the Eye for Students and General Practitioners. By Charles H. May, M.D., chief of clinic and instructor in ophthalmology, College of Physicians and Surgeons, Medical Department of Columbia University, 1890-1893; attending ophthalmic surgeon to the Mt. Sinai Hospital. Sixth edition. Revised with

362 original illustrations, including 22 plates with 62 colored figures. New York: William Wood & Company. 1909. Price, $2.00, net.)

The Practical Medicine Series. Comprising ten volumes on the year's progress in medicine and surgery, under the general charge of Gustavus P. Head, M.D., professor laryngology and rhinology at the Chicago Postgraduate Medical School. Vol. IV., Gynecology. Edited by Emilius C. Dudley, A.M.,M.D., professor of gynecology Northwestern University Medical School and C. von Bachellé, M.S., M.D., assistant professor of obstetrics Chicago Polyclinic and College of Physicians and Surgeons. Series, 1909. Chicago: The Year Book Publishers. (Price, 1.25.)

The Malarial Fevers, Hemoglobinuric Fever and the Blood Protozoa of Man. By Charles F. Craig, M.D., Captain Medical Corps United States Army, attending surgeon New York; late pathologist and bacteriologist to the Sternberg U. S. Army General Hospital. Chicamauga Park, Ga. Illustrated by four colored plates, twenty-five clinical charts and twenty-eight photomicrographs and drawings. New York: William Wood & Company. 1909. (Price, $4.50.)

Manual of Therapeutics referring especially to the products of the Pharmaceutical and Biological Laboratories of Parke, Davis & Company. Detroit: Parke, Davis & Company. 1909.

Medical Jurisprudence, Forensic Medicine and Toxicology. Prepared by numerous experts of acknowledged authority under the supervision and editorship of R. A. Witthaus, M.D., professor of chemistry, medical jurisprudence and toxicology Cornell University and Tracy C. Becker, A.B., LL.B., professor of general law and medical jurisprudence, University of Buffalo. Second edition. Volume III. New York: William Wood & Company. 1909. (Price, $6.00 a volume.)

Hudson-Fulton Celebration, 1609—1807—1909. September 25 to October 9, 1909. A brochure issued for the use of the schools of the state. Compiled and edited by Harlan Hoyt Horner. Albany: New York State Education Department. 1909.

